# Paeoniflorin alleviates 17α-ethinylestradiol-induced cholestasis *via* the farnesoid X receptor-mediated bile acid homeostasis signaling pathway in rats

**DOI:** 10.3389/fphar.2022.1064653

**Published:** 2022-11-21

**Authors:** Rulin Wang, Tengteng Yuan, Jing Sun, Menghuan Yang, Yunna Chen, Lei Wang, Yanyan Wang, Weidong Chen, Daiyin Peng

**Affiliations:** ^1^ College of Pharmacy, Anhui University of Chinese Medicine, Hefei, Anhui, China; ^2^ Anhui Province Key Laboratory of Chinese Medicinal Formula, Hefei, Anhui, China; ^3^ Department of Pharmacy, The First Affiliated Hospital of Anhui Medical University, Hefei, Anhui, China; ^4^ College of Chinese Medicine, Anhui University of Chinese Medicine, Hefei, Anhui, China

**Keywords:** paeoniflorin (Pae), cholestasis, 17α-ethinylestradiol, bile acid, UHPLC-MS/MS, farnesoid X receptor (FXR)

## Abstract

Cholestasis, characterized by disturbance of bile formation, is a common pathological condition that can induce several serious liver diseases. As a kind of trigger, estrogen-induced cholestasis belongs to drug-induced cholestasis. Paeoniflorin is the most abundant bioactive constituent in *Paeonia lactiflora* Pall., *Paeonia suffruticosa* Andr., or *Paeonia veitchii* Lynch, a widely used herbal medicine for treating hepatic disease over centuries in China. However, the pharmacologic effect and mechanism of paeoniflorin on estrogen-induced cholestasis remain unclear. In this experiment, the pharmacological effect of paeoniflorin on EE-induced cholestasis in rats was evaluated comprehensively for the first time. Ultra-high-performance liquid chromatography coupled with Q-Exactive orbitrap mass spectrometer was used to monitor the variation of bile acid levels and composition. It was demonstrated that paeoniflorin alleviated 17α-ethinylestradiol (EE)-induced cholestasis dose-dependently, characterized by a decrease of serum biochemical indexes, recovery of bile flow, amelioration of hepatic and ileal histopathology, and reduction of oxidative stress. In addition, paeoniflorin intervention restored EE-disrupted bile acid homeostasis in enterohepatic circulation. Further mechanism studies using western blot, quantitative Real-Time PCR, and immunohistochemical showed that paeoniflorin could upregulate hepatic efflux transporters expression but downregulate hepatic uptake transporter expression. Meanwhile, paeoniflorin reduced bile acids synthesis by repressing cholesterol 7α-hydroxylase in hepatocytes. Paeoniflorin affected the above transporters and enzyme *via* activation of a nuclear receptor, farnesoid X receptor (FXR), which was recognized as a vital regulator for maintaining bile acid homeostasis. In conclusion, paeoniflorin alleviated EE-induced cholestasis and maintained bile acid homeostasis *via* FXR-mediated regulation of bile acids transporters and synthesis enzyme. The findings indicated that paeoniflorin might exert a potential therapeutic medicine for estrogen-induced cholestasis.

## 1 Introduction

Cholestatic liver disease is a clinically pathological condition characterized by abnormal bile flow, always accompanied by the accumulation of intrahepatic and extrahepatic bile acids (Fernandez-Murga et al., 2018). Many factors can evoke cholestasis, including hepatitis, gene mutations, metabolic disorders, and drugs (Gijbels et al., 2019). As a drug-induced cholestasis, estrogen-induced cholestasis mainly occurs in vulnerable women taking oral contraceptives or receiving postmenopausal hormone replacement therapy (Bach et al., 2020). 17α-ethinylestradiol (EE), one of the synthetic estrogen derivatives, is extensively employed in research on estrogen-induced cholestasis (Marrone et al., 2016). Ursodeoxycholic acid (UDCA) is the most widely prescribed medicine for cholestasis, gallstones, and fatty liver disease (Li et al., 2016). However, about 40% of patients with cholestasis show poor responses to UDCA treatment (Hirschfield et al., 2015). Consequently, it is essential to develop novel therapeutic medicine for cholestasis.

During cholestasis development, bile excretion disorder is the most intuitive pathological manifestation, along with the variation of endogenous bile acids composition and proportion (Hirschfield et al., 2010). In hepatocytes, primary bile acids are synthesized from cholesterol through enzymatic reactions (Šarenac and Mikov, 2018) and subsequently excreted by transporters on the membranes of hepatocytes (Phelps et al., 2019). After being transported to the capillary duct between hepatocytes and combined with amino acids (Hua et al., 2021), the primary bile acids enter the small intestine through the bile duct with bile flow (Bogatyrev et al., 2020). Primary bile acids are converted to secondary bile acids by the action of intestinal flora (Jiao et al., 2018). Most of the bile acids in the intestine are absorbed into the portal circulation through bile acid transporters distributed on intestinal epithelial cells (Tiratterra et al., 2018), and then enter the hepatocytes through uptake transporters on the hepatocyte membrane (Bowman et al., 2019), a process also known as the enterohepatic circulation of bile acids (Sun et al., 2021b). Normally, the composition and proportion of bile acids are in a stable state (Setchell et al., 1997), but when homeostasis is disrupted, various pathological disorders in the hepatoenteric system may occur, including cholestasis (Zhang et al., 2008). Bile discharge disorders also lead to the accumulation of toxic bile acids (Oizumi et al., 2017). Therefore, targeting endogenous bile acids contributes to understanding the influence of cholestasis and medicine treatment on bile acid homeostasis.

Bile acids can act as ligands to activate various nuclear receptors, including the farnesoid X receptor (FXR, NR1H4), which belongs to a subclass of metabolic receptors within the nuclear receptor superfamily (Keitel et al., 2019). FXR is mainly located in the liver and intestine (Sun et al., 2021a) and plays a vital role in regulating bile acids homeostasis (Trauner et al., 2017). Activation of FXR has been shown to inhibit cholesterol 7α-hydroxylase (CYP7A1), a key rate-limiting enzyme in the classical bile acid synthesis pathway (Chiang and Ferrell, 2020). FXR could induce transporters, such as multidrug resistance-associated protein 2 (MRP2) and bile salt export pump (BSEP), to reinforce bile acids efflux while inhibiting Na^+^-dependent taurocholate cotransporter (NTCP) from reducing bile acids uptake by hepatocytes (Stofan and Guo, 2020). Targeting FXR activity has emerged as a novel strategy to treat cholestasis and other hepatic diseases (Kowdley et al., 2018; Hirschfield et al., 2019; Trauner et al., 2019). Some FXR agonists, such as obeticholic acid, have been approved by the FDA for the treatment of primary biliary cirrhosis in patients who are intolerant to or non-responsive to first-line therapy (van Golen et al., 2018).

Paeoniflorin (Supplementary Figure S1A) is the most abundant bioactive constituent in *Paeonia lactiflora* Pall., *Paeonia suffruticosa* Andr., or *Paeonia veitchii* Lynch, which is one of the most widely used herbal medicine for hepatic disease over 2,000 years in China (Ma et al., 2020). It has been well established that paeoniflorin has numerous pharmacological effects on hepatic diseases, such as hepatic ischemia/reperfusion alleviation (Xie et al., 2018), cholestasis alleviation (Wei et al., 2020), hepatic fibrosis attenuation (Wang et al., 2021), nonalcoholic fatty hepatic disease prevention (Ma et al., 2016), and so on. Crucially, paeoniflorin has been shown to remarkably alleviate alpha-naphthylisothiocyanate (ANIT)-induced cholestatic hepatitis (Zhao et al., 2017; Zhou et al., 2017; Chen et al., 2021). However, the pharmacology effect of paeoniflorin on EE-induced cholestasis has not been thoroughly evaluated.

The present study explored whether paeoniflorin had a valuably alleviated effect on EE-induced cholestasis in rats and further clarified whether this effect is related to the regulation of bile acid transporters *via* FXR, which affected endogenous bile acid homeostasis. The findings prove that paeoniflorin might become a potential candidate for cholestasis.

## 2 Materials and methods

### 2.1 Materials

Paeoniflorin (Lot: CFN99544) was obtained from ChemFaces (Wuhan, China), and the purity was proven to be over 98%. EE (Lot: E2014109), UDCA (Lot: I2016160), and standard for taurodeoxycholic acid (TDCA) (Lot: K1922115) were purchased from Aladdin Biochemical Technology (Shanghai, China). Standards for cholic acid (CA) (Lot: 100,078–201415), deoxycholic acid (DCA) (Lot: 110,724–200207), UDCA (Lot: 110,755–201704) chenodeoxycholic acid (CDCA) (Lot: 110,806–201507), hyodeoxycholic acid (HDCA) (Lot: 100,087–201411), taurocholic acid (TCA) (Lot: 110,815–201510), tauroursodeoxycholic acid (TUDCA) (Lot: 110,816–201509), and taurochenodeoxycholic acid (TCDCA) (Lot: 110,846–201007) were obtained from National Institutes for Food and Drug Control (Beijing, China). Standards for *β*-muricholic acid (β-MCA) (Lot: 700233P-1MG-A-010) and tauro-β-muricholic acid (T-β-MCA) (Lot: 700244P-1 MG-B-010) were purchased from Avanti^®^ Polar Lipids (AL, United States). The standard for lithocholic acid (LCA) (Lot: FCB055902) was acquired from Fluorochem (Derbyshire, United Kingdom). The standard for internal standard (IS), dehydrocholic acid (dhCA) (Lot: 3CMJG-S), was purchased from TCI (Tokyo, Japan). Antibodies directed against FXR, NTCP, CYP7A1, MRP2, and *β*-actin were acquired from Bioss (Beijing, China), BSEP was purchased from Santa Cruz Biotechnology (CA, United States), and liver X receptor α (LXRα, NR1H3) was purchased from Abcam (Cambridge, United Kingdom). All chemical reagents were analytical or HPLC grade.

### 2.2 Animal experiments

Male Sprague-Dawley rats (5 weeks, 220 ± 20 g) were acquired from the Experimental Animal Center of Anhui Medical University (Hefei, China). Rats were housed in standard conditions of temperature and humidity. All animal experimental procedures were implemented in keeping with international guidelines and approved by the Institutional Animal Care and Use Committee, Anhui University of Chinese Medicine.

After one week of adaptive feeding, rats were randomly divided into seven groups (n = 8 per group): control group, only paeoniflorin (200 mg/kg) administration group, model (EE) group, paeoniflorin-treated (100, 200, 400 mg/kg) groups, and UDCA-treated (100 mg/kg) group as the positive control. As shown in Supplementary Figure S1B, rats were orally administrated with paeoniflorin, UDCA, or normal saline twice daily for seven consecutive days. Since the 3^rd^ day, model, paeoniflorin-treated (100, 200, 400 mg/kg), and UDCA-treated groups were subcutaneously injected with EE (10 mg/kg), while control and only paeoniflorin administration groups were subcutaneously injected with vehicle (propylene glycol) once a day for five consecutive days. After overnight fasting, all rats were sacrificed on the 7^th^ day. Serum, liver, and ileum were collected.

### 2.3 Bile flow measurement

Before the experimental operation, rats were anesthetized with 3% pentobarbital sodium (30 mg/kg body weight). For bile flow measurement, rats were received a middle abdominal incision, and then the common bile duct was cannulated with PE-10 polyethylene tubes at 37°C for 1 h to collect the bile. Later, the bile volume was gravimetrically determined with a 1.0 g/ml density.

### 2.4 Serum biochemical analyses

The serum levels of alanine aminotransferase (ALT), aspartate aminotransferase (AST), alkaline phosphatase (ALP), total bile acid (TBA), total bilirubin (TBIL), direct bilirubin (DBIL), and γ-glutamyl transferase (γ-GT) were determined by commercial kits which were purchased from Jiancheng Bioengineering Institute (Nanjing, China). The practice was according to the manufacturer’s guidelines.

### 2.5 Measurement of oxidative stress indexes in rat livers

Liver tissues were ground to homogenize with 0.9% saline (1:9, w:v) on ice, then the homogenate was transferred to the centrifugation (3,000 rpm, 20 min, 4°C), and the supernatant was retained. The involvement of oxidative stress was assessed by measuring the levels of malondialdehyde (MDA) and superoxide dismutase (SOD) in liver homogenate, using rat-specific enzyme-linked immunosorbent assay (ELISA) kits obtained from Dogesce (Beijing, China).

### 2.6 Histopathology

Samples from liver and ileum tissues were fixed in formalin, embedded in paraffin, sectioned, and then stained with hematoxylin and eosin (H&E). Images were captured by Olympus microscope (Tokyo, Japan) to evaluate tissue structural changes.

### 2.7 Quantitative profiling of bile acids in rat serum, bile, and liver using ultra-high-performance liquid chromatography coupled to hybrid orbitrap mass spectrometry system

#### 2.7.1 Sample preparation

For serum samples, 500 μl acetonitrile was added to 50 μl rat serum which was accurately spiked with 10 μl dhCA (IS). The mixture was vortexed and centrifuged at 12,000 rpm and 4°C for 10 min. The upper layer was collected and evaporated to dryness under a vacuum. The residue was redissolved in a 200 μL mobile phase, vortexed for 3 min, and centrifuged at 12,000 rpm and 4°C for 10 min. The supernatant was transferred to another tube and centrifuged again, and then it was collected and filtered through a 0.22 μm membrane. An aliquot (2 μL) was injected into the ultra-high-performance liquid chromatography (UHPLC) system for analysis.

For bile samples, 10 μl rat bile was diluted with 490 μl deionized water and mixed well. Then, a 50 μl diluted bile sample was accurately spiked with 10 μl IS, followed by the addition of 500 μL acetonitrile. Subsequent treatment was the same as that of the serum samples.

For liver samples, 50 mg of rat liver samples were homogenized in 500 μl normal saline. 100 μl of liver homogenate was accurately spiked with 10 μl IS, and 1 ml of acetonitrile was added. Subsequent treatment was the same as that of the serum samples.

#### 2.7.2 Ultra-high-performance liquid chromatography coupled to hybrid orbitrap mass spectrometry system conditions

Liquid chromatography was carried out by a Dionex Ultimate 3000 XRS UHPLC system (Thermo Fisher Scientific). Separation was performed on a Hypersil GOLD^™^ C18 column (2.1*100 mm, 1.9 μm, Thermo Fisher Scientific), and the column oven temperature was kept at 30°C. The mobile phase consists of ultrapure water with 5 mM ammonium acetate (solvent A) and methanol (solvent B). A gradient elution procedure was used as follows: 0–2 min, 60% B; 2–18 min, 60%–64% B; 18–19 min 64%–95% B; 19–21 min, 95% B; 21–22 min, 95%–60% B; 22–24 min, 60% B. The constant flow rate was set at 0.2 ml/min. The injection volume was 2 μl. Typical chromatograms are shown in Supplementary Figure S2.

Mass spectrometry (MS) detection was implemented on a high-resolution hybrid quadrupole Q-Exactive Orbitrap MS (Thermo Fisher Scientific), preceded by heated electrospray ionization (HESI). The mass spectrometer was operated in parallel reaction monitor (PRM) mode. The sheath gas flow rate, auxiliary gas flow rate, and sweep gas flow rate were set to 45 psi, 15 psi, and 1 psi, respectively. The heater and capillary temperature were both set at 350°C, and the spray voltage for negative ionization was 3.1 kV. The PRM transitions and MS parameters for individual bile acids and IS in the Q-Exactive Focus Orbitrap MS method are shown in Supplementary Table S1.

Regression equations, correlation coefficient, and linear ranges for individual bile acids are shown in Supplementary Table S2.

### 2.8 Quantitative Real-Time PCR assay

Total RNA from rat liver samples was extracted by TRIzol reagent (Ambion, Austin, United States). 1 μg of total RNA in each sample was reverse-transcribed into cDNA using SPARKscript Ⅱ RT Plus Kit (With gDNA Eraser) (Sparkjade, Qingdao, China). The mRNA expression of the target gene was quantified by 2 ×SYBR Green qPCR Mix (With ROX) (Sparkjade, Qingdao, China). The expression of rat *β-actin* was used as the internal reference. Relative gene expression was detected in triplicate using the ABI StepOne Plus system (Applied Biosystems, CA, United States). The primer sequences used in the present study are listed in Supplementary Table S3.

### 2.9 Western blot analysis

Total protein samples in the liver tissues of rats were extracted by RIPA lysis buffer (Beyotime Biotechnology, China). The protein concentration was measured using a BCA protein assay kit (Beyotime Biotechnology, China). 30 μg protein in each liver sample was resolved using 6%–12% SDS-PAGE and then transferred onto nitrocellulose filter membranes. Membranes were blocked and then incubated with primary antibodies directed against FXR, LXRα, NTCP, BSEP, MRP2, CYP7A1, and *β*-actin overnight at 4°C. After rinsing with TBST, the membranes were subsequently incubated with horseradish peroxidase-conjugated goat anti-rabbit/mouse secondary antibody for 1.5 h at room temperature. Protein bands were detected on Amersham Imager 600 (GE Healthcare, United States) or BLT GelView 6000Plus (Guangzhou Biolight Biotechnology, Ltd., China) with enhanced chemiluminescence detection reagents (Thermo, United States).

### 2.10 Immunohistochemistry

The protein expression of FXR in liver and ileum tissues were detected by immunohistochemistry. Paraffin sections were pretreated with antigen retrieval and then immersed in 3% H_2_O_2_ for 20 min at room temperature. The sections were incubated with primary antibody directed against FXR (1:200) for 60 min at 37°C. Subsequent operations followed the laboratory routine procedures. Images were captured by an Olympus microscope (Tokyo, Japan).

### 2.11 Statistical analysis

The data were expressed as the mean ± standard deviation (SD). Statistics were implemented using the GraphPad Prism 8 software with additional analysis in IBM SPSS Statistics 25. Differences between the two groups were analyzed by unpaired student’s *t*-test, and multiple group comparisons were performed using one-way analysis of variance. Statistical significance was set to *p* < 0.05.

## 3 Results

### 3.1 Paeoniflorin protected against EE-induced hepatotoxicity

**FIGURE 1:**
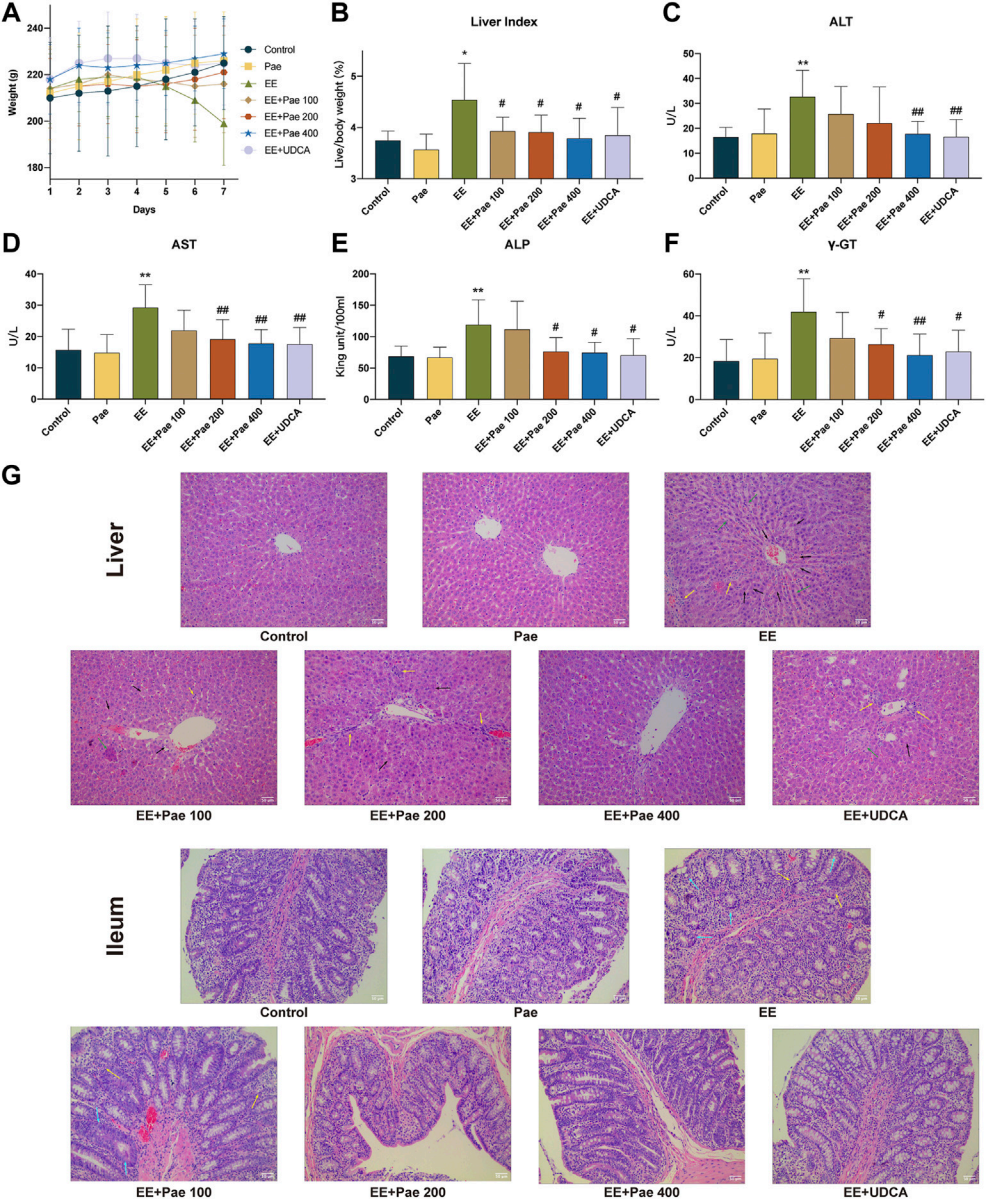
Hepatoprotective effects of paeoniflorin on EE-induced cholestatic hepatic injury. **(A)** Trends of body weight change during the seven days of administration. **(B)** Liver index. The serum levels of **(C)** ALT, **(D)** AST, **(E)** ALP, and **(F)** γ-GT in each group. **(G)** The images of liver and ileum sections with H&E (× 200 magnification). Yellow arrows indicate the aggregation of inflammatory cells, black arrows indicate hepatocyte pyknosis and necrosis, green arrows indicate the disordered arrangement of the hepatic cords, and blue arrows indicate structural defects of intestinal villi or glands. Data are expressed as the mean ± SD. (*n* = 8). *p < 0.05, **p < 0.01, versus control group; ^#^ *p* < 0.05, ^##^ *p* < 0.01, versus EE group.

As shown in Figure 1A, the body weight of the control and only paeoniflorin-treated group gradually increased, while that of EE-treated rats fell. After treatment with paeoniflorin, the growth retardation caused by EE was reduced in a dose-dependent manner. As shown in Figure 1B, liver index (Liver weight/body weight) showed an opposite tendency compared to body weight gain. Administration of paeoniflorin and UDCA relieved EE-induced hepatomegaly.

Compared to the control group, the serum levels of ALT, AST, ALP, and γ-GT were significantly higher in EE-treated groups. However, as illustrated in Figures 1C–F, high-dose paeoniflorin (400 mg/kg) could significantly decrease the abnormal ALT, AST, ALP, and γ-GT levels in EE-induced cholestatic rats. Medium-dose paeoniflorin (200 mg/kg) could significantly reduce the abnormal AST ALP, and γ-GT levels in cholestatic rats, but has no significant effect on ALT. Low-dose paeoniflorin (100 mg/kg) had no significant effect on hepatotoxicity biochemical indicators. Besides, administration of UDCA, the positive control in the study, reduced body weight loss and down-regulated serum biochemical indexes in EE-treated rats.

As shown in Figure 1G, the H&E staining results indicated that EE induced hepatocyte pyknosis and necrosis, inflammatory cells aggregation, hepatic cord arrangement disorder, hepatic sinus dilatation and hyperemia. The hepatic structure disorder was improved in rats treated with paeoniflorin or UDCA. On the other hand, defective intestinal mucosal epithelium, villi, and gland structure were observed in the EE-treated group. There were also clusters of inflammatory cells in certain areas of the ileum. The structure of ileum was improved after treatment with medium- or high-dose paeoniflorin and UDCA.

### 3.2 Paeoniflorin ameliorated bile flow and biochemical indicators of cholestasis

**FIGURE 2:**
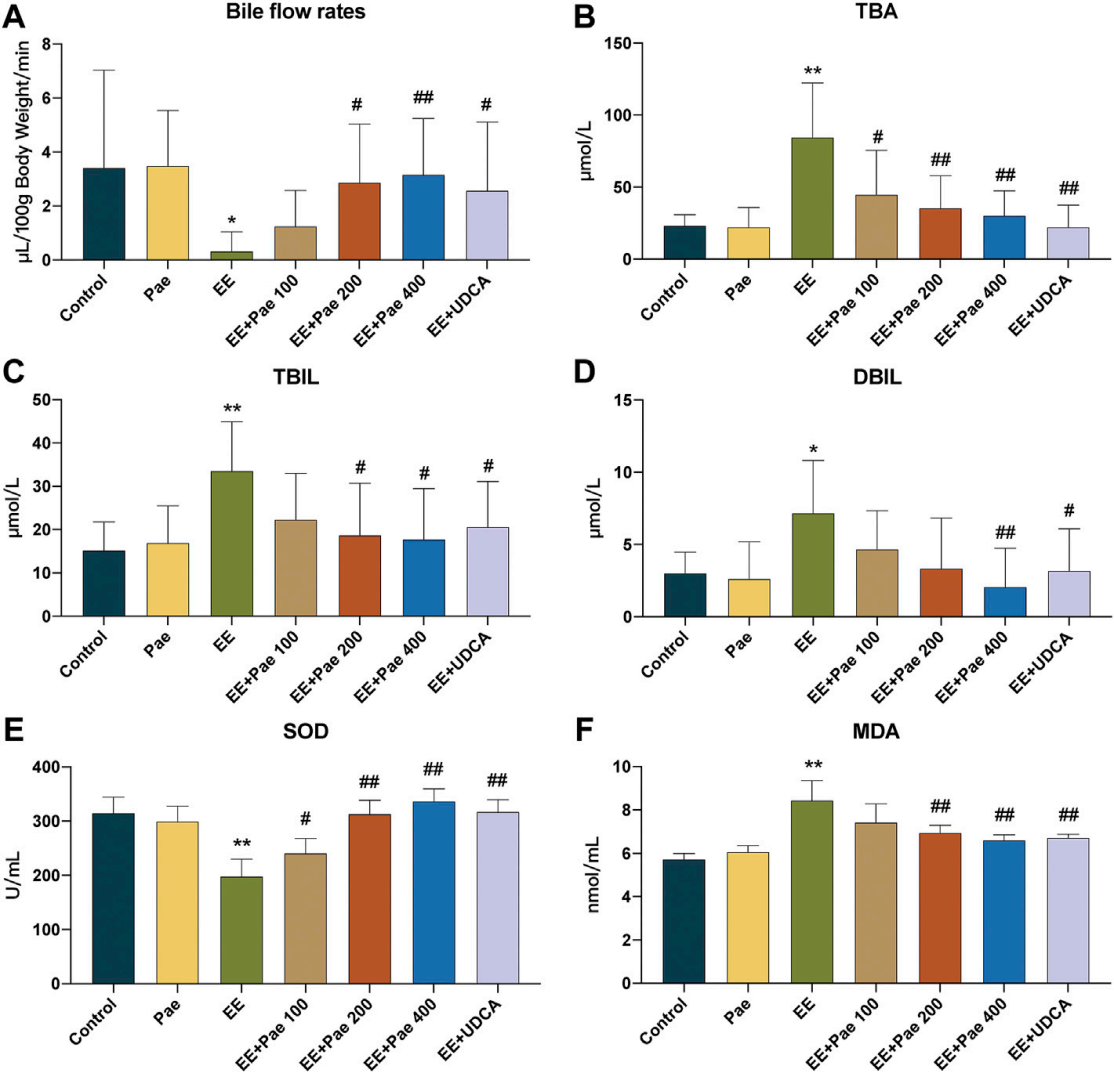
Paeoniflorin attenuated EE-induced cholestasis in rats. **(A)** The bile flow rates over 1 h. The serum levels of **(B)** TBA, **(C)** TBIL, and **(D)** DBIL in each group (mean ± SD, n = 8). Levels of **(E)** SOD and **(F)** MDA in liver tissue homogenate (mean ± SD, *n* = 6). *p < 0.05, **p < 0.01, versus control group; ^#^ *p* < 0.05, ^##^ *p* < 0.01, versus EE group.

Bile flow in rats was observed over 60 min. As reported, EE-treated rats showed significant bile flow obstruction. Medium- and high-dose paeoniflorin administration remarkably increased bile flow rates compared to the model group (Figure 2A). As shown in Figures 2B–D, the serum TBA, TBIL, and DBIL were increased in EE-treated group compared to the control group. In contrast, these biochemical indicators of cholestasis were all reduced by high-dose paeoniflorin treatment. Medium-dose paeoniflorin significantly decreased the levels of TBA and TBIL, while low-dose paeoniflorin only had effect on TBA.

### 3.3 Paeoniflorin improved EE-induced oxidative stress

Oxidative stress is an important driving force in promoting cholestasis. The level of hepatic SOD, the antioxidant enzyme, was decreased in EE-treated rats (Figure 2E), while the level of hepatic MDA, the biomarker for oxidative damage, was increased (Figure 2F). However, paeoniflorin and UDCA treatment significantly reversed these trends. These findings collectively prove that paeoniflorin improved EE-induced oxidative stress.

### 3.4 Paeoniflorin maintained bile acid homeostasis in cholestatic rats

The concentrations of free and taurine-conjugated bile acids in serum, bile, and liver samples were determined by the UHPLC-Orbitrap MS method.

#### 3.4.1 Effect of paeoniflorin on bile acid levels in serum

**FIGURE 3:**
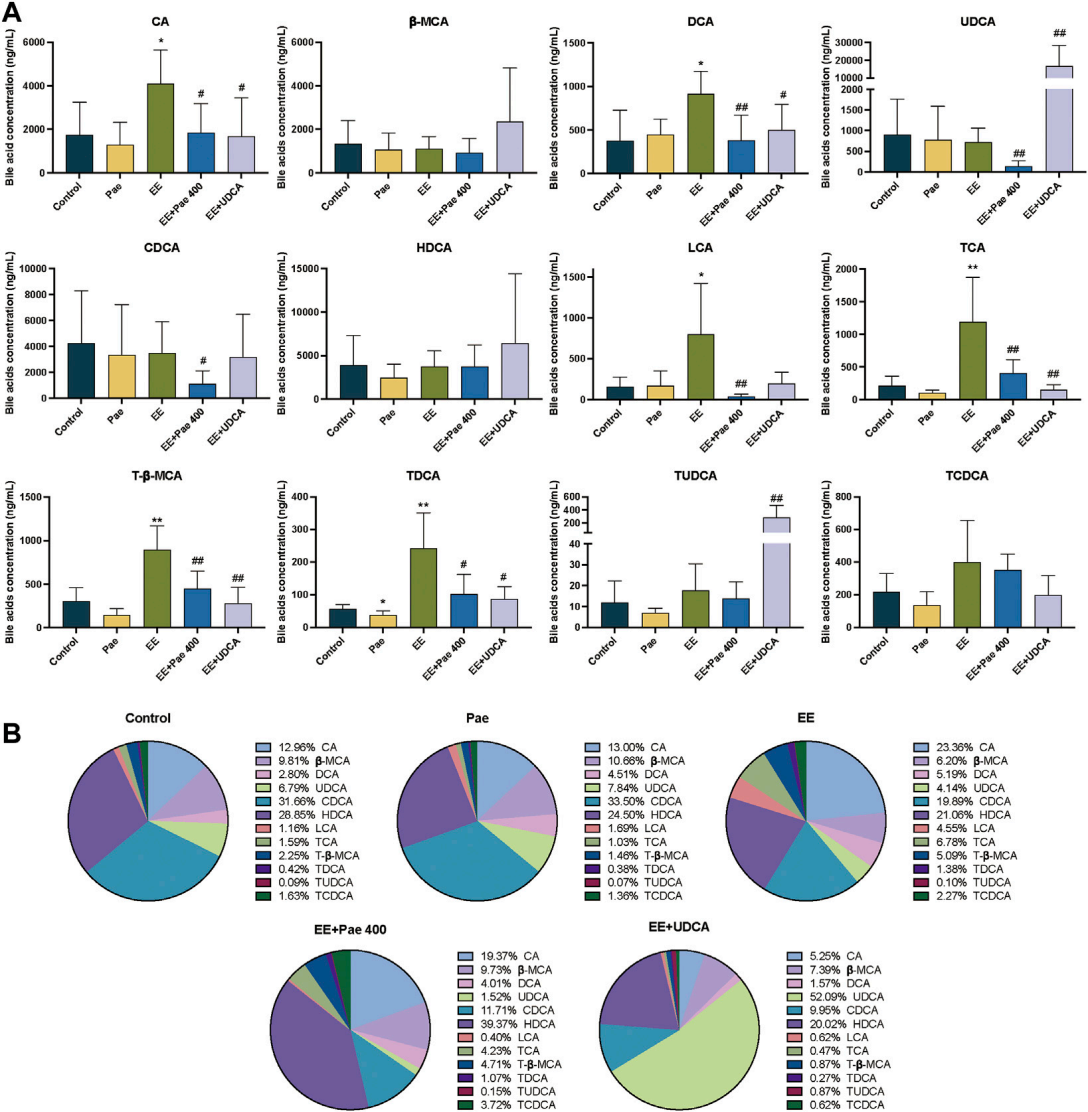
Paeoniflorin improved EE-induced abnormal serum bile acid levels and composition. **(A)** Concentrations of individual bile acids in serum of different groups (Mean ± SD, *n* = 6). *p < 0.05, **p < 0.01, versus control group; ^#^ *p* < 0.05, ^##^ *p* < 0.01, versus EE group. **(B)** Bile acids composition in serum (Mean/Total×100%, *n* = 6).

Free bile acids accounted for more than 85% of the TBA in serum. As shown in Figure 3A, EE treatment significantly increased the serum levels of CA, DCA, LCA, TCA, T-β-MCA, and TDCA compared to the control group. The proportion of the above bile acids were increased in the EE-treated group, while *β*-MCA, UDCA, and HDCA were decreased (Figure 3B). Compared to the EE-treated group, the serum levels of CA, DCA, UDCA, CDCA, LCA, TCA, T-β-MCA, and TDCA were significantly decreased after paeoniflorin intervention. The proportion of bile acid components in the paeoniflorin intervention group was closer to the level of the control group except for UDCA, CDCA and T-β-MCA. When UDCA was given to rats, the proportion of UDCA in serum elevated rapidly to about 50%, while the proportion of other bile acids declined. The concentrations of UDCA and TUDCA were significantly increased while CA, DCA, TCA, T-β-MCA, and TDCA were decreased considerably.

#### 3.4.2 Effect of paeoniflorin on bile acid levels in bile

**FIGURE 4:**
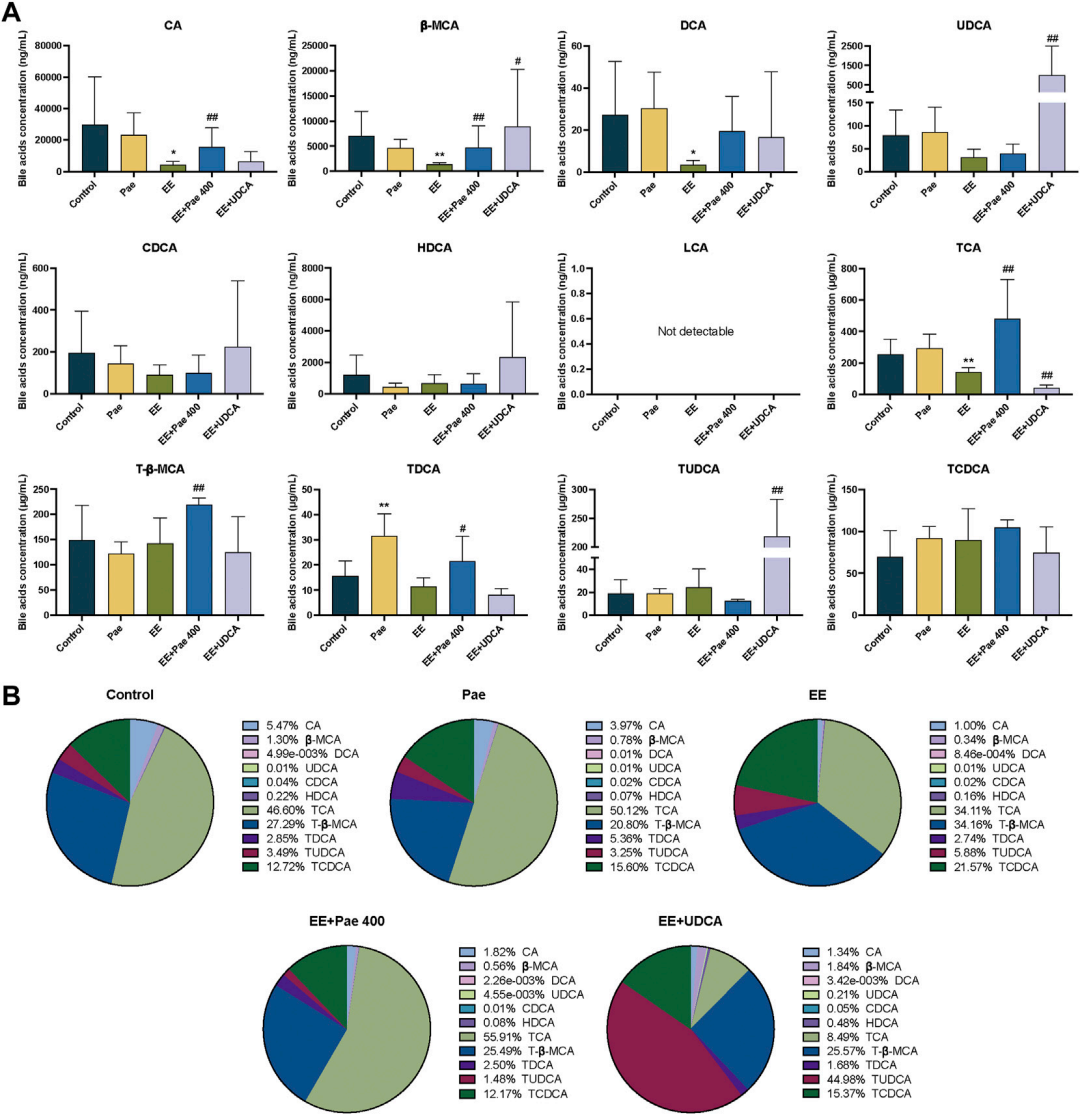
Paeoniflorin improved EE-induced abnormal bile acid levels and composition in bile. **(A)** Concentrations of individual bile acids in bile of different groups (Mean ± SD, *n* = 6). *p < 0.05, **p < 0.01, versus control group; ^#^ *p* < 0.05, ^##^ *p* < 0.01, versus EE group. **(B)** Bile acids composition in bile (Mean/Total × 100%, *n* = 6).

The bile acids in bile were mainly conjugated type, taurine-conjugated bile acids accounted for more than 92% in each group in the current study. As shown in Figure 4A, compared to the control group, the concentrations of CA, *β*-MCA, DCA, and TCA in the model group were significantly decreased. For the composition, the proportion of CA and TCA was decreased, while the proportion of T-β-MCA and TCDCA was elevated (Figure 4B). That is, when cholestasis occurs, the CA and TCA secreted by bile are significantly reduced, indicating that these bile acids may be stored in the liver. Compared to the EE-treated group, the concentrations of CA, *β*-MCA, TCA, T-β-MCA, and TDCA in the paeoniflorin intervention group were significantly increased. Regarding bile acid composition, the proportion of bile acid was closer to the level of the control group in the paeoniflorin administration group except for CA. When UDCA was given to rats, the proportion of TUDCA was remarkably increased. The concentrations of *β*-MCA, UDCA, and TUDCA were significantly elevated after UDCA intervention, while the level of TCA was decreased.

#### 3.4.3 Effect of paeoniflorin on intrahepatic bile acid levels

**FIGURE 5:**
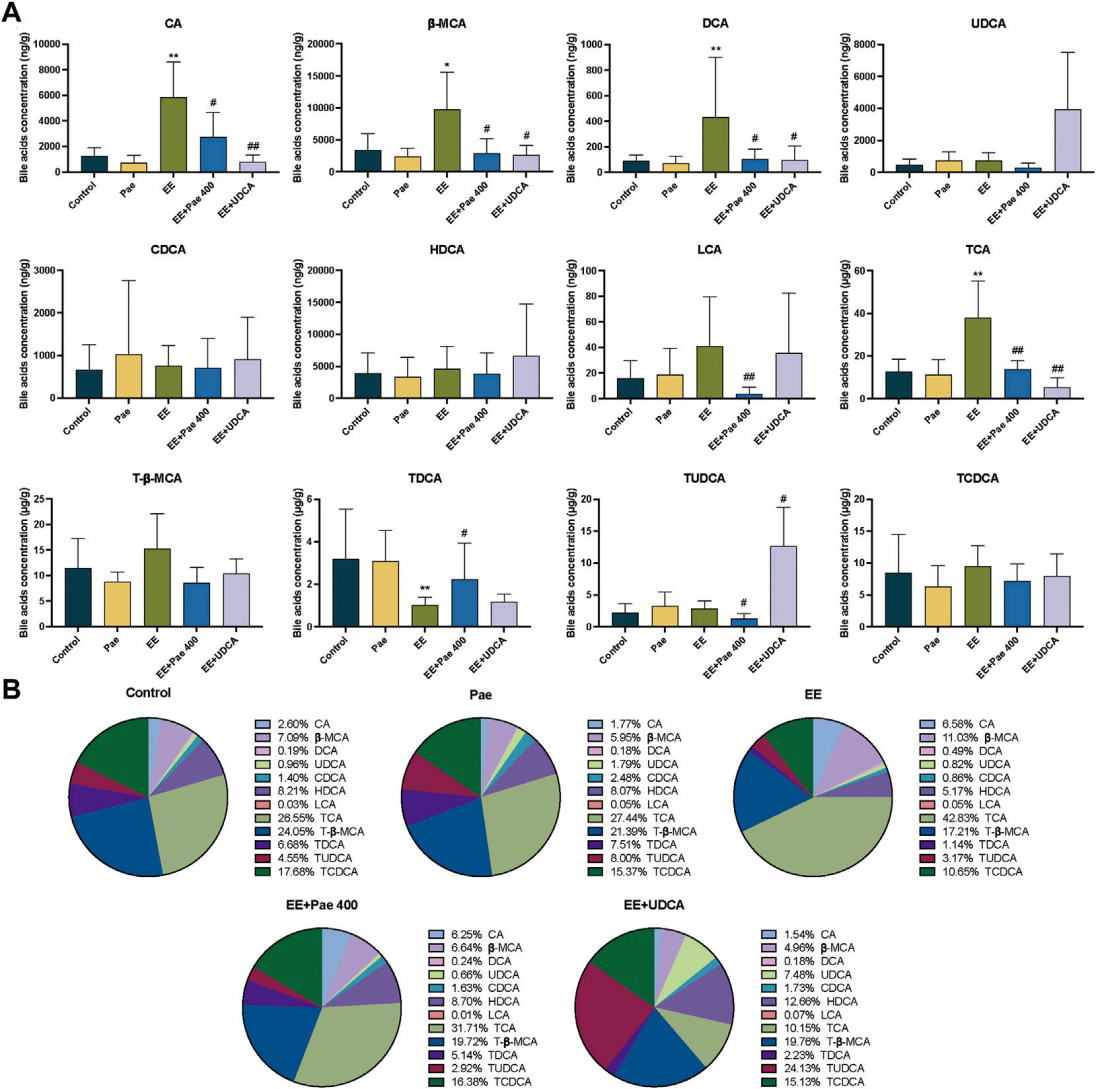
Paeoniflorin improved EE-induced abnormal intrahepatic bile acid levels and composition. **(A)** Concentrations of hepatic individual bile acids in different groups (Mean ± SD, *n* = 6). *p < 0.05, **p < 0.01, versus control group; ^#^ *p* < 0.05, ^##^ *p* < 0.01, versus EE group. **(B)** Hepatic bile acids composition (Mean/Total × 100%, *n* = 6).

Intrahepatic bile acids were mainly conjugated type, and taurine-conjugated bile acids accounted for more than 70% in each group in this experiment. As shown in Figure 5A, compared to the control group, the concentrations of CA, *β*-MCA, DCA, and TCA in the model group were significantly increased, while concentration of TDCA was decreased. For bile acid composition, the proportion of CA, *β*-MCA, and TCA was increased, while the proportion of CDCA, HDCA, T-β-MCA, TDCA, and TCDCA was decreased (Figure 5B). Compared to the model group, intrahepatic concentrations of CA, *β*-MCA, DCA, LCA, TCA, and TUDCA in the paeoniflorin intervention group were significantly decreased, while the concentration of TDCA was significantly increased. In terms of composition, the proportions of *β*-MCA and TCA were decreased significantly, while CDCA, HDCA, T-β-MCA, TDCA, and TCDCA were elevated. Besides, the proportions of bile acids were closer to the level of the control group except for CA. When UDCA was administered to rats, the level and proportion of TUDCA were remarkably increased. The concentrations of CA, *β*-MCA, DCA, and TCA were significantly descended after UDCA intervention, while the level of TUDCA was increased.

### 3.5 Paeoniflorin activated FXR and LXRα expression in cholestatic rats

**FIGURE 6:**
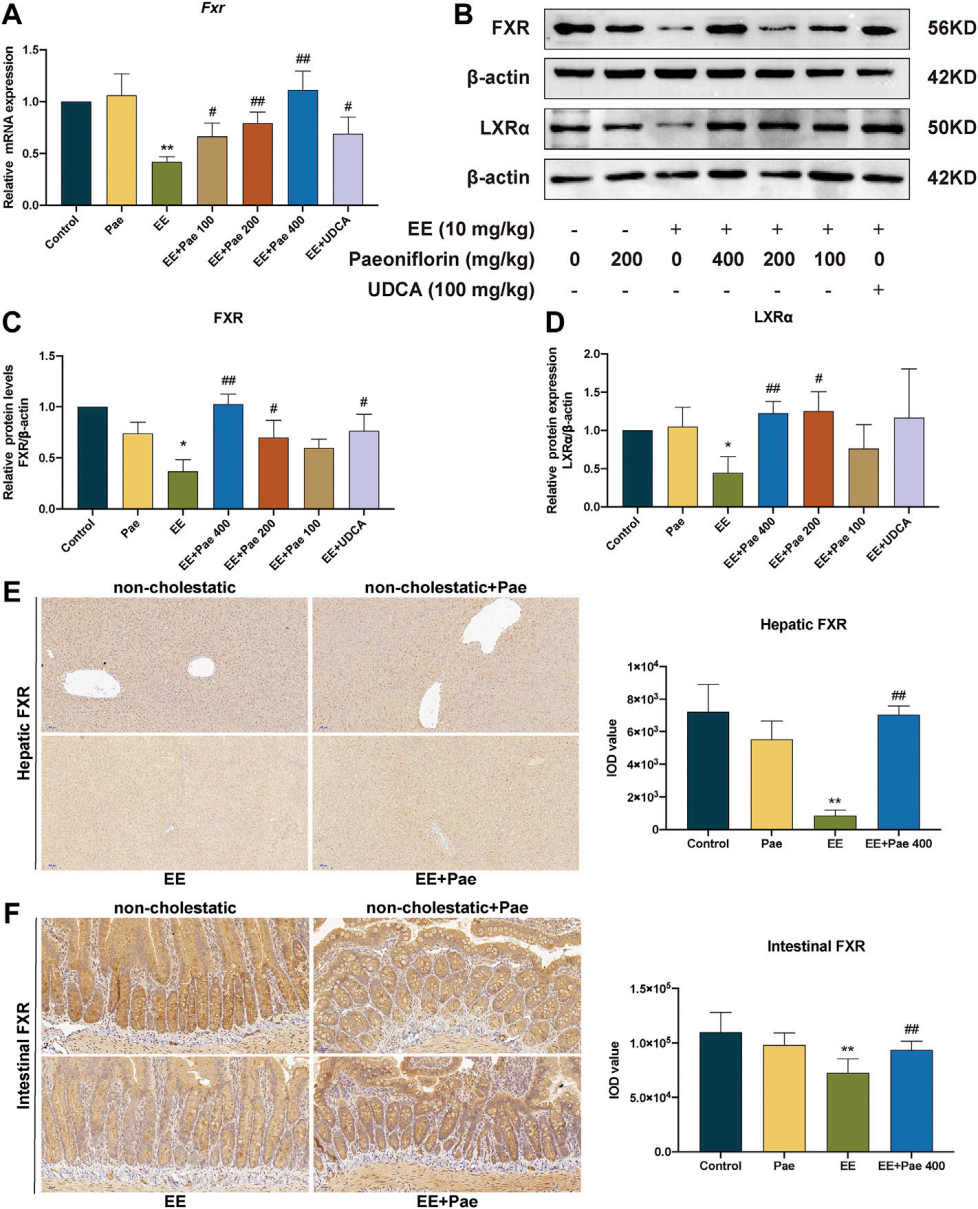
Paeoniflorin restored the expression of FXR and LXRα. **(A)** The mRNA expression of nuclear receptor *Fxr*, was normalized to *β-actin*. **(B)** The western blot images of FXR and LXRα. The protein expression of **(C)** FXR, and **(D)** LXRα, were normalized to *ß*-actin. **(E)** Representative immunohistochemical images of FXR in rat liver. **(F)** Representative immunohistochemical images of FXR in rat ileum. Data are expressed as the mean ± SD. (*n* = 3 for mRNA and protein expression; *n* = 6 for FXR expression in immunohistochemical images). *p < 0.05, **p < 0.01, versus control group; ^#^ *p* < 0.05, ^##^ *p* < 0.01, versus EE group.

Liver nuclear receptor FXR plays a crucial role in maintaining intrahepatic bile acids homeostasis *via* mediating synthesis, metabolism, and transport of bile acids. As shown in Figure 6A, compared to the control group, the quantitative real-time PCR assay (qRT-PCR) results showed that EE-treated group significantly reduced the mRNA expression of FXR, and paeoniflorin treatment significantly reversed this effect. Western blot analysis further confirmed the effects of paeoniflorin (Figures 6B,C). Administration of UDCA also showed an up-regulation with mRNA and protein expression of FXR.

Immunohistochemical staining demonstrated that expression of hepatic and intestinal FXR was suppressed by EE induction, while improved by high-dose paeoniflorin administration. This suggested that paeoniflorin administration could activate both hepatic and intestinal FXR expression (Figures 6E,F).

In addition to FXR, LXRα is another nuclear receptor proven as a physiological regulator of cholesterol and lipid metabolism, which disturbed sensitivity to bile acid toxicity and cholestasis. Compared to the control group, the protein expression of LXRα was decreased in the EE-treated group (Figures 6B,D). Treatment with medium- and high-dose paeoniflorin significantly increased LXRα expression. However, the positive control, UDCA, did not considerably affect LXRα expression.

### 3.6 Paeoniflorin regulated the expression of hepatobiliary bile acids transporters and bile acids synthetic enzyme

**FIGURE 7:**
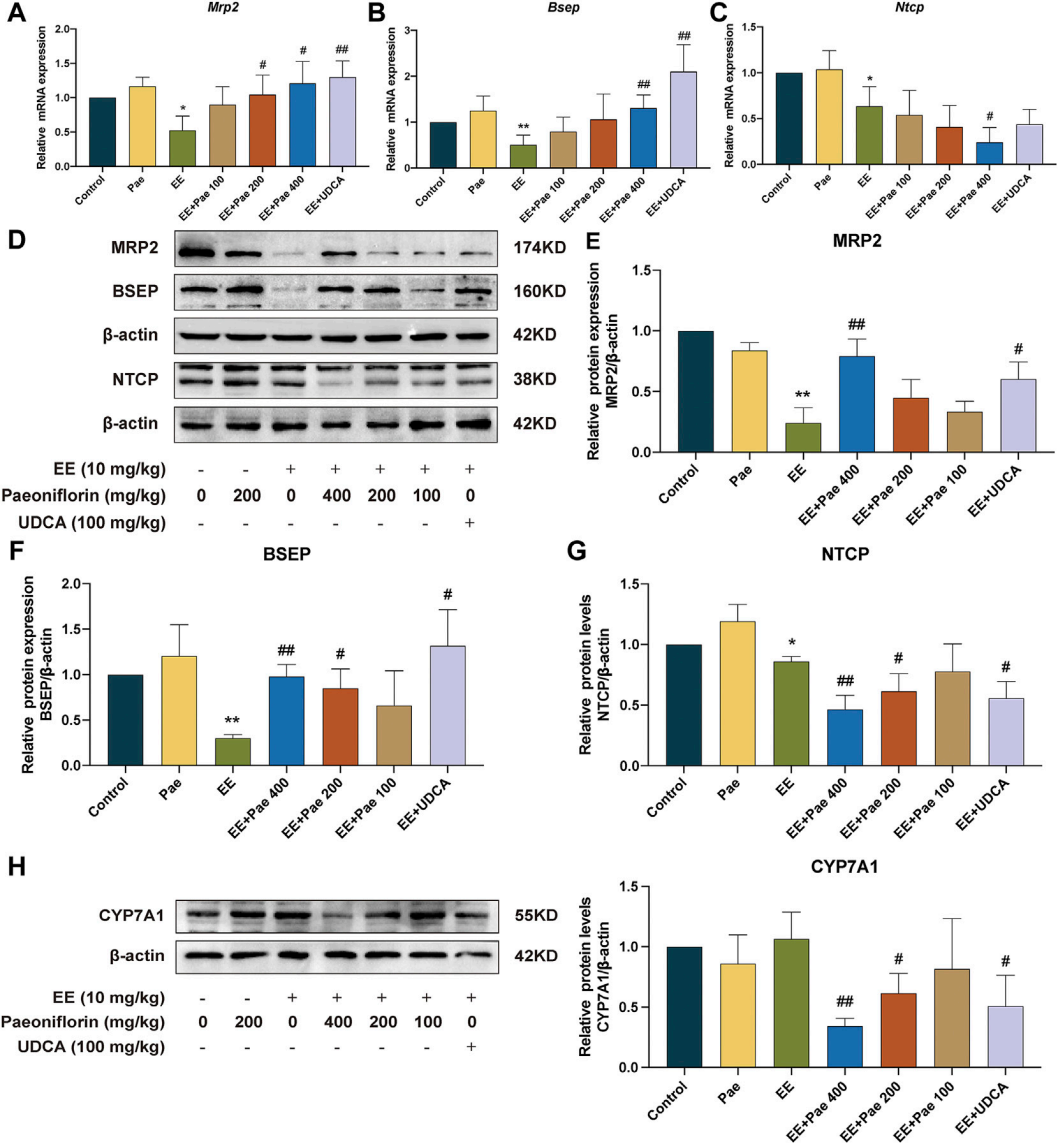
Paeoniflorin promoted bile acid efflux transporters, while reduced bile acid influx transporter and bile acid synthesis enzyme in rats. Relative mRNA expression of **(A)** *Mrp2*, **(B)** *Bsep*, and **(C)** *Ntcp*, were normalized to *β-actin*. **(D)** The western blot images of MRP2, BSEP, NTCP, and *ß*-actin. The protein expression of **(E)** MRP2, **(F)** BSEP, and **(G)** NTCP, were normalized to *ß*-actin. **(H)** The protein expression of CYP7A1 was determined by western blot and normalized to *ß*-actin. Data are expressed as the mean ± SD. (*n* = 3). *p < 0.05, **p < 0.01, versus control group; ^#^ *p* < 0.05, ^##^ *p* < 0.01, versus EE group.

To illustrate the mechanism of paeoniflorin mitigating cholestasis, the present study determined the mRNA and protein expression of hepatobiliary bile acid transporters, including BSEP, MRP2, and NTCP. These above transporters are proven as downstream targets of FXR in previous reports. As shown in Figures 7A–C, EE treatment significantly decreased the mRNA expression of *Bsep* and *Mrp2*, both of which play critical roles in hepatic bile acid efflux. High-dose paeoniflorin intervention significantly increased the mRNA expression of these transporters. Besides, the mRNA expression of *Ntcp*, which is known as a basolateral uptake transporter, showed a similar downward trend after EE-induced cholestasis. However, the mRNA expression of *Ntcp* was further reduced by high-dose paeoniflorin administration. Western blot analysis further confirmed these qRT-PCR results (Figures 7D–G). Besides, administration of UDCA increased the mRNA and protein expression of BSEP and MRP2. In contrast, it suppressed the protein expression of NTCP but had no statistically significant effect on the mRNA expression of *Ntcp*.

CYP7A1 is a critical rate-limiting enzyme in the conversion process from cholesterol to bile acids. As shown in Figure 7H, compared to the EE-treated group, both 200 and 400 mg/kg paeoniflorin administration dose-dependently down-regulated CYP7A1 expression, contributing to a decrease in bile acid synthesis. The positive drug, UDCA, also decreased the protein expression of CYP7A1.

## 4 Discussion

Endogenous and exogenous estrogens, including their metabolites, would result in intrahepatic cholestatic liver injury in susceptive women who exhibited abnormal bile flow and hepatitis (Padda et al., 2011). As a hepatotoxic substance, EE has been widely applied in rodents to explore underlining mechanisms of estrogen-induced cholestatic hepatic diseases, which belongs to drug-induced cholestatic hepatic disorders (Yang et al., 2019; Ming et al., 2021). Previous reports have proven that EE injection reduced bile flow and led to cholestasis *via* the FXR-meditated Extracellular regulated protein kinases (ERK)-Liver kinase B1 (LKB1)-Adenosine 5′-monophosphate-activated protein kinase (AMPK) pathway (Li et al., 2017). To illuminate the pharmacological activity of paeoniflorin on EE-induced cholestasis and explore whether the effect is relevant to FXR, the current study adopted a rodent model in Sprague-Dawley rats injected with EE once daily for five successive days. In this animal model, EE-induced cholestasis with a defect of pivotal canalicular solute transporters of bile acids, including BSEP, MRP2, and NTCP (Marrone et al., 2016). Currently, UDCA is the first-line clinical medicine for cholestasis, so it is applied as the positive control in the current study. Previous reports have fully demonstrated the improved effect of UDCA administration on EE-induced cholestatic liver injury (Li et al., 2016; Di Guida et al., 2018).

Paeoniflorin is the primary active component in *Paeonia lactiflora pall*, which is extracted from the dried root of the plants*.* Previous studies have demonstrated that paeoniflorin exerted effectively protective effects on various hepatic diseases (Tu et al., 2019), including ANIT-induced cholestasis (Zhao et al., 2013; Chen et al., 2015; Chen et al., 2021) and bile duct ligation-induced cholestatic liver injury (Wei et al., 2020). This study fully elucidated that paeoniflorin showed an alleviative effect on EE-induced hepatotoxicity for the first time, as characterized by significant reduction of hepatotoxicity biochemical serum markers, including ALT, AST, ALP, and γ-GT, which were elevated by EE induction. Moreover, ameliorative hepatic and ileal histopathology was observed in paeoniflorin-treated group.

Cholestasis is mainly manifested as decelerated bile flow. In this study, injection of EE significantly decreased bile flow and elevated cholestatic biochemical indicators, including TBIL, DBIL, and TBA. Administration of paeoniflorin or UDCA could significantly improve these trends. In the development of cholestasis, oxidative stress is an important driving force that cannot be ignored. The results indicated that the MDA level was elevated while the SOD level was declined in the liver of EE-treated rats, indicating that oxidative stress was activated. The present study showed that paeoniflorin could restore indicators of oxidative stress. Overall, paeoniflorin alleviated EE-induced cholestasis, which was characterized by increasing bile flow, decreasing serum biomarkers, ameliorating hepatic and ileal histopathology, and improving oxidative stress.

Bile acid is one of the major ingredients in bile. In the enterohepatic system, bile acids can facilitate the assimilation of lipids, fat-soluble vitamins, and cholesterol in the intestine (Lee et al., 2020). Destabilization of bile acid homeostasis may trigger various pathological changes, including cholestasis (Fickert and Wagner, 2017). Moreover, when cholestasis occurs, the body accumulates a large quantity of bile, leading to hepatocellular necrosis with the turbulence of phospholipids, unsaturated fatty acids, and sphingolipids metabolism, which further aggravates hepatic diseases (Lin et al., 2019). Compared to the control group, the levels of key bile acids in serum were markedly elevated after EE induction, indicating that the uptake function of bile acid by hepatocytes was damaged. Meanwhile, the levels of CA, TCA, *β*-MCA, and DCA were significantly increased in the liver while considerably decreased in the bile, indicating that the function of the bile acid efflux transporter on the hepatocyte membrane is disturbed, leading to the reduction of bile acids efflux into bile and accumulation in the liver. Among bile acids with abnormal levels, LCA and DCA are considered hepatotoxic, and their accumulation exacerbates hepatocyte injury caused by cholestasis (Chen et al., 2014; Li et al., 2016). Administration of paeoniflorin in cholestatic rats could maintain sensitive endogenous bile acids in the serum, bile, and liver at relatively stable levels, indicating that the bile acid homeostasis was maintained when bile excretion was promoted by paeoniflorin. Since bile acid homeostasis is mainly regulated by bile acid transporters on the hepatocyte membrane, it can be speculated that paeoniflorin has a regulatory effect on bile acid transporters, which is inseparable from the influence of upstream nuclear receptors of the transporters.

Nuclear receptors have been demonstrated to regulate target gene expression in numerous metabolic processes, including bile acid synthesis and conjugation, through ligand-activated transcription (Beuers et al., 2015). FXR is a crucial regulator in the maintenance of bile acid homeostasis (Gomez-Ospina et al., 2016), as FXR activation restores the levels and metabolic abnormalities of bile acids pool and exerts therapeutic effects on cholestasis in rodents (Dong et al., 2019). In contrast, specific FXR deficiency would lead to the reduction of bile flow. Nowadays, several FXR agonists are undergoing clinical trials for various types of cholestasis treatment (Kowdley et al., 2018; Trauner et al., 2019). The present experiment explored whether FXR was involved in the alleviation of paeoniflorin against EE-induced cholestasis. The results illustrated that EE induced a significant decrease in FXR expression, and paeoniflorin administration could restore the mRNA and protein expression of FXR in a dose dependent manner. As a weak FXR agonist, UDCA administration upregulated EE-suppressed FXR expression at both mRNA and protein levels.

LXRα is distributed in the liver, kidney, and intestine, known as metabolically active receptor. Both FXR and LXRα can form heterodimers with the retinoid X receptors and heterodimers in the active state bound to the response element on the DNA in the basal state (Hiebl et al., 2018). According to the analysis of mammalian one-hybrid and transient transfection reporters, paeoniflorin has been found to activate LXRα in HepG2 cells (Lin, 2013). In this study, it was observed that EE injection down-regulated LXRα expression while over 200 mg/kg paeoniflorin could markedly increase LXRα expression at the protein level. In contrast, UDCA intervention showed no significant influence on LXRα.

Bile acid transporters and metabolic enzymes are essential for maintaining bile acid homeostasis. As two crucial bile acid transporters on the hepatocyte membrane, BSEP and MRP2 regulate the transport of free and conjugated bile acids into bile. EE treatment significantly decreased the mRNA and protein expression of BSEP and MRP2, leading to bile flow obstruction and bile acids accumulation. BSEP and MRP2 expression were significantly increased by paeoniflorin administration, promoting hepatic bile acid efflux. NTCP regulates the transition of conjugated bile acid uptake into hepatocytes from the portal venous blood. The expression of NTCP was reduced in EE-induced cholestasis, which contributed to the relief of cholestasis. Previous studies have shown that estrogen-induced cholestasis reduced basolateral organic anion-transporting polypeptides, such as NTCP and OATPs (Geier et al., 2003). Nonetheless, the decreases of BSEP and MRP2, which are mainly responsible for canalicular secretion of bile acids, played dominant roles in EE-induced cholestasis. Paeoniflorin and UDCA administration further reduced NTCP expression, contributing to the improvement of cholestatic liver injury.

As a bile acid synthetic enzyme, CYP7A1 plays a crucial role in bile acid synthesis. By activating the hepatic small heterodimer partner, FXR can repress CYP7A1 to reduce bile acid synthesis in hepatocytes (Chiang et al., 2000). The present study revealed that paeoniflorin down-regulated the protein level of CYP7A1 in a dose-dependent manner. Nonetheless, decreased bile acid synthesis enzyme in cholestatic rats might be an adaptive response to reduce drug-induced hepatotoxicity.

## 5 Conclusion

**FIGURE 8:**
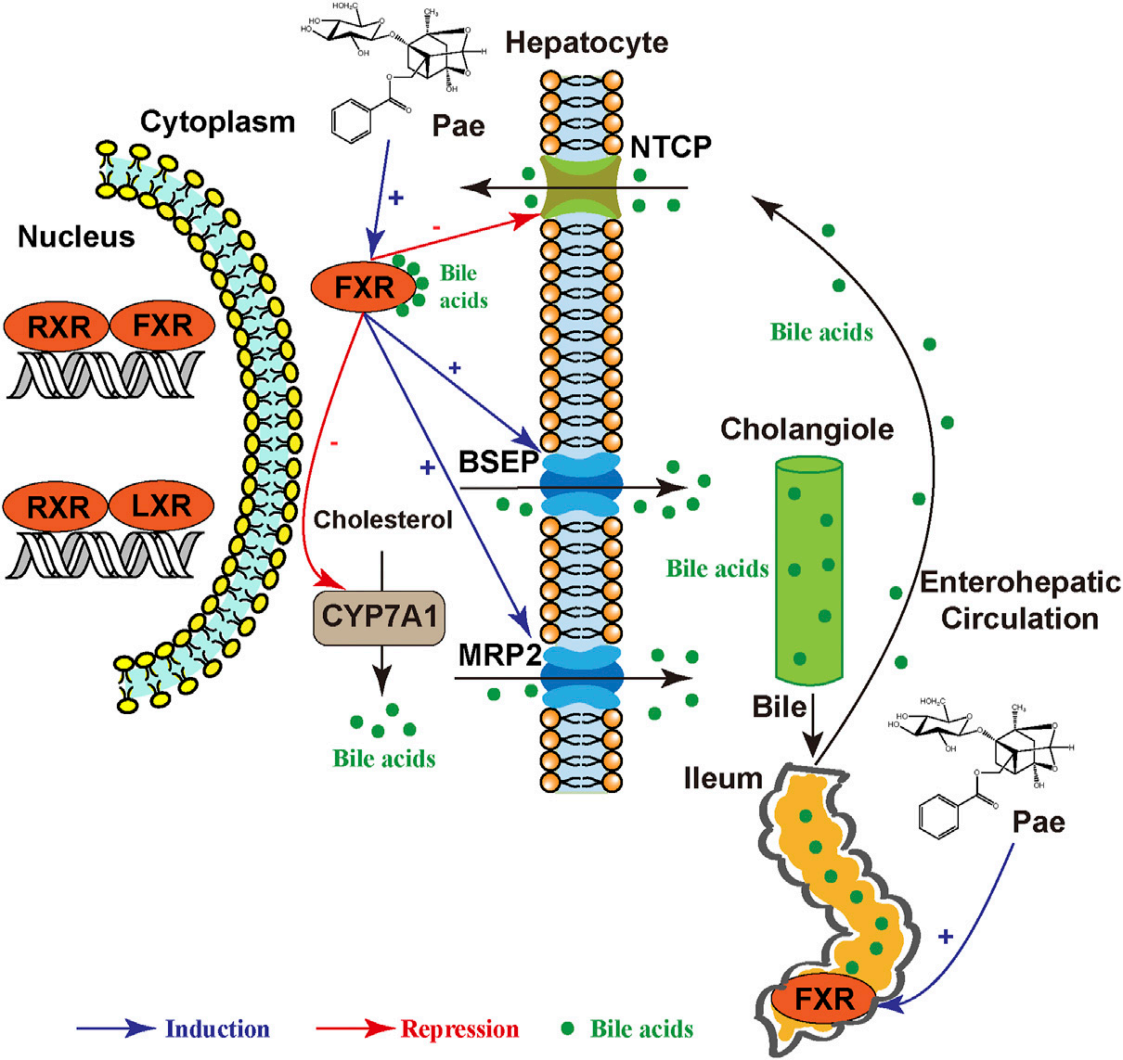
The possible mechanisms of paeoniflorin on alleviating estrogen-induced cholestasis.

In summary, the present study demonstrated that paeoniflorin dose-dependently alleviated EE-induced cholestasis and maintained bile acid homeostasis in rats. Moreover, the results also indicated a correlation between nuclear receptors, bile acid transporters, synthetic enzyme, and bile acid homeostasis with the alleviation effect of paeoniflorin on EE-induced cholestasis. The impact of paeoniflorin against cholestasis may account for the activation of the FXR-mediated bile acid homeostasis signaling pathway, which contributes to upregulation of bile acids efflux and downregulation of bile acids uptake and synthesis (Figure 8). The present study suggested that paeoniflorin may become a potential therapeutic agent for estrogen-induced cholestasis.

## Data Availability

The original contributions presented in the study are included in the article/Supplementary Material, further inquiries can be directed to the corresponding authors.
