# Supplementary material for: Paeoniflorin alleviates 17α-ethinylestradiol-induced cholestasis via the farnesoid X receptor-mediated bile acid homeostasis signaling pathway in rats
Source: Front Pharmacol. 2022 Nov 21;13:1064653. doi: 10.3389/fphar.2022.1064653 (PMC9719974; doi:10.3389/fphar.2022.1064653)
Supplement: Supplementary file 1 [file DataSheet1.docx]

Supplementary Material


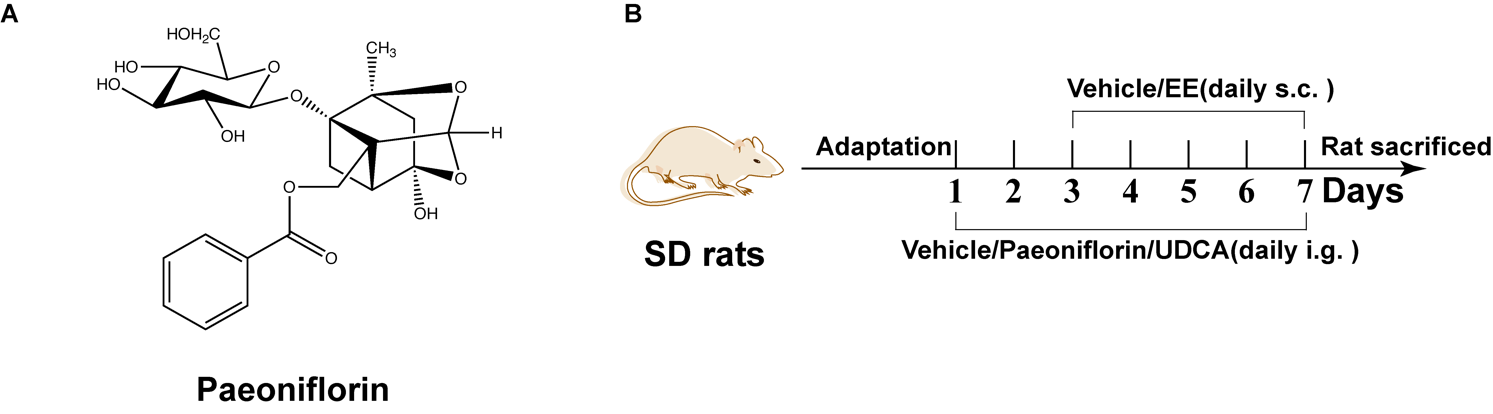


**Supplementary Figure 1.** **(A)** The chemical structure of paeoniflorin; **(B)** Schematic of the animal experiments.

**Supplementary Figure 2-1.** Typical chromatogram of blank serum.


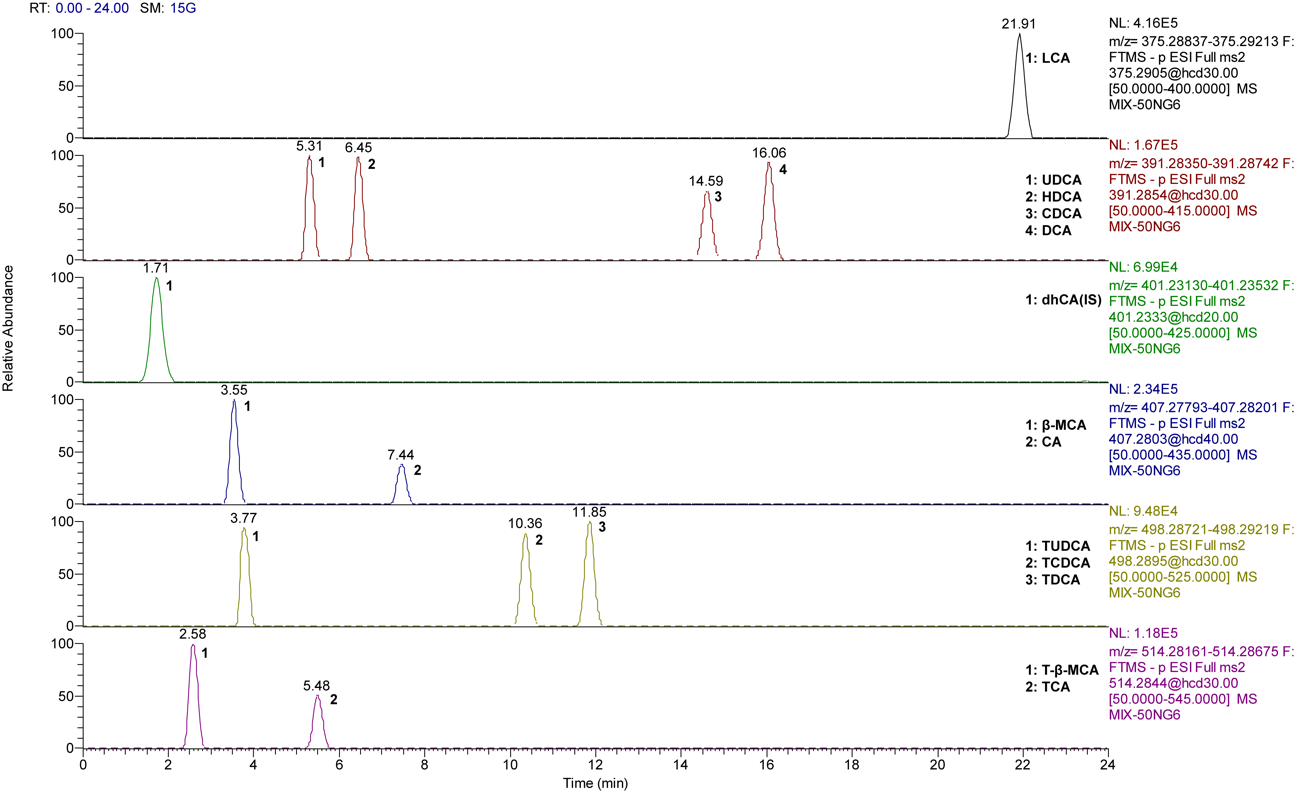


**Supplementary Figure 2-2.** Typical chromatogram of working solution containing 12 fully identified bile acids and IS without matrix.


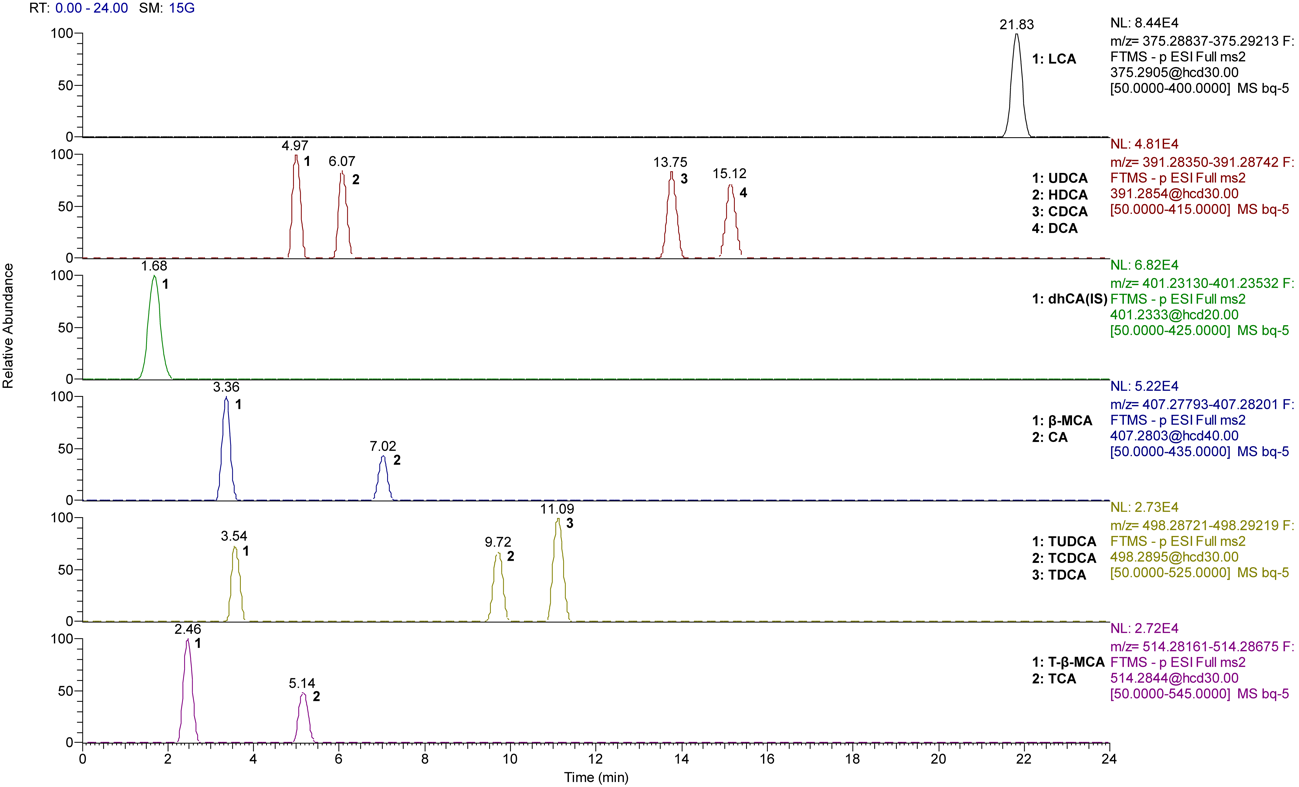


**Supplementary Figure 2-3.** Typical chromatogram of blank serum which spiked with 12 fully identified bile acids and IS.


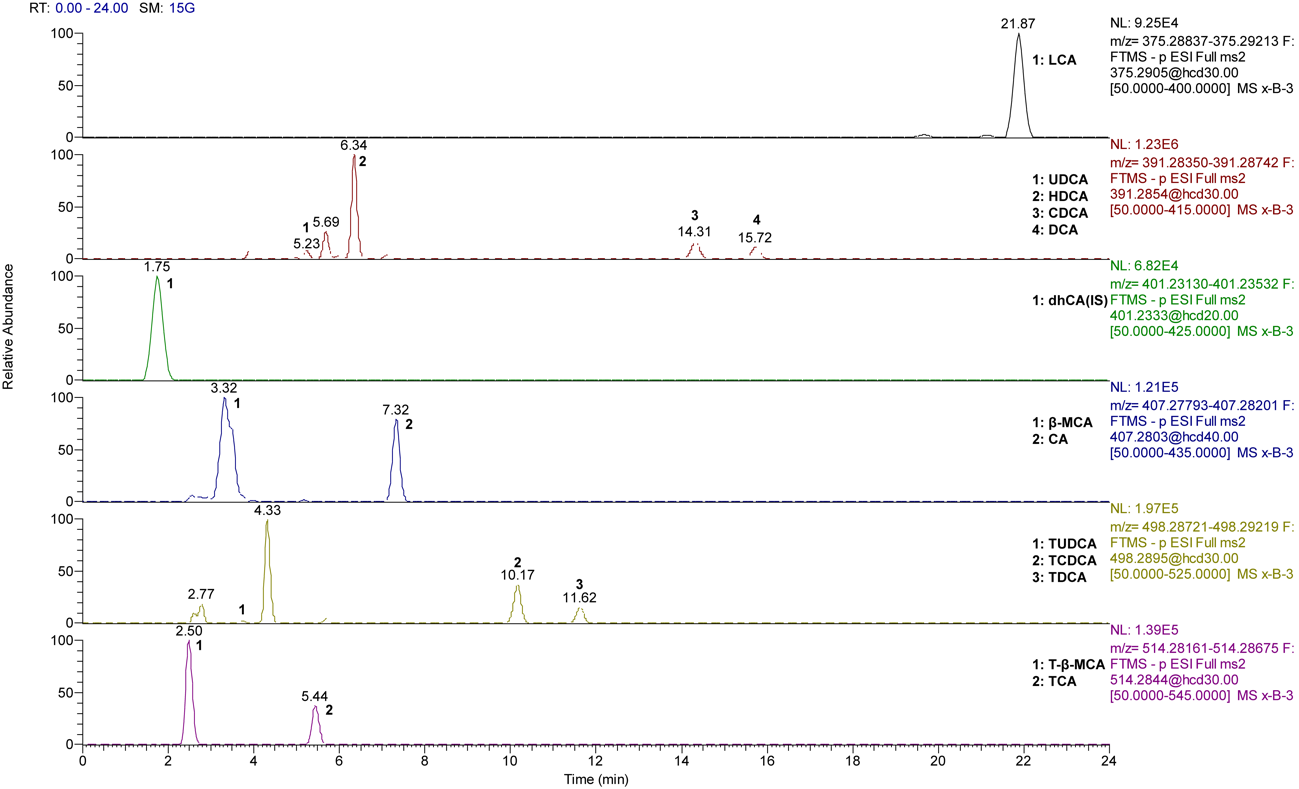


**Supplementary Figure 2-4.** Typical chromatogram of the serum sample which spiked with IS.


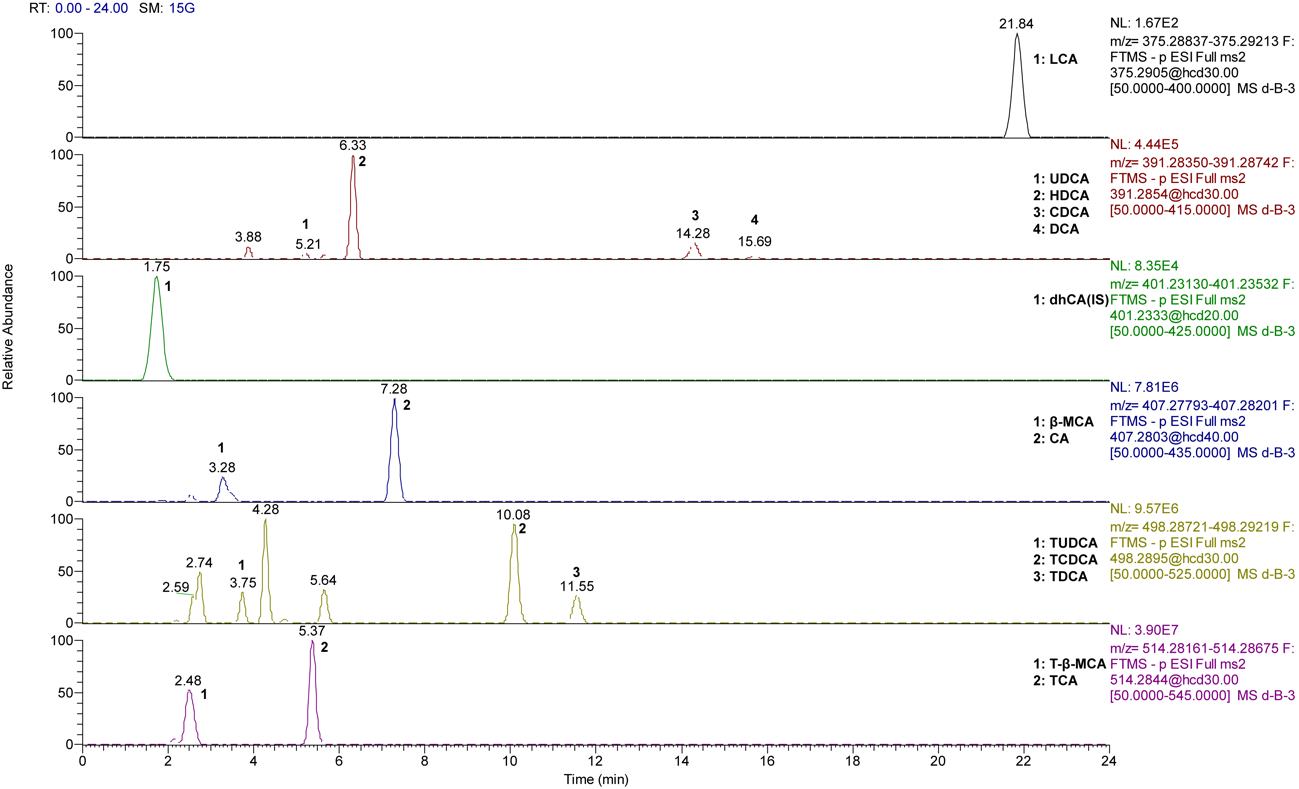


**Supplementary Figure 2-5.** Typical chromatogram of the bile sample which spiked with IS.


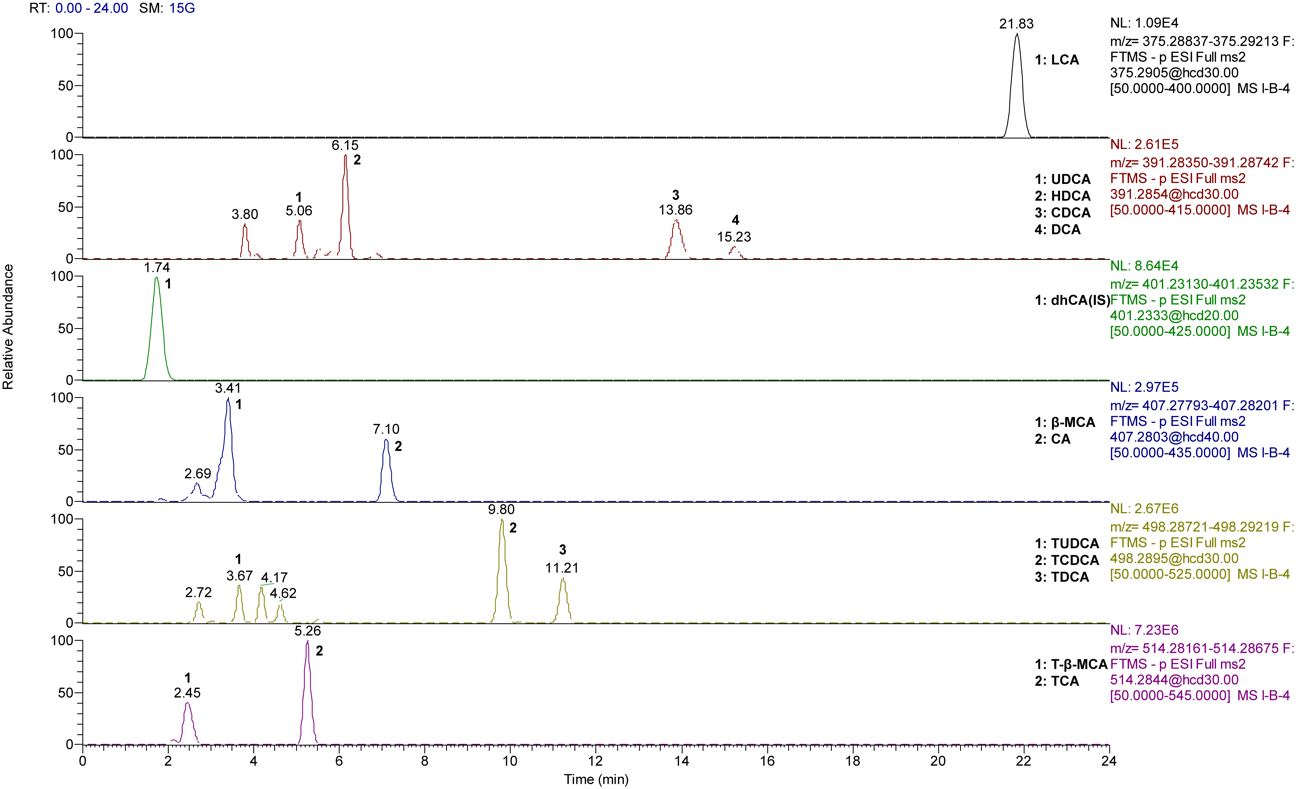


**Supplementary Figure 2-6.** Typical chromatogram of the liver sample which spiked with IS.

**Supplementary Table 1** PRM transitions and mass spectrometry parameters for individual bile acids and IS in the Q-Exactive Focus Orbitrap MS method

| **Compound** | **Precursor (m/z)** | **Product (m/z)** | **Polarity** | **(N)CE** | **Retention Time (min)** |
| --- | --- | --- | --- | --- | --- |
| CA | 407.28030 | 407.27699 | Negative | 40 | 7.44 |
| β-MCA | 407.28030 | 407.27699 | Negative | 40 | 3.55 |
| DCA | 391.28538 | 391.28500 | Negative | 30 | 16.06 |
| UDCA | 391.28538 | 391.28500 | Negative | 30 | 5.31 |
| CDCA | 391.28538 | 391.28500 | Negative | 30 | 14.59 |
| HDCA | 391.28538 | 391.28500 | Negative | 30 | 6.45 |
| LCA | 375.29047 | 375.29034 | Negative | 30 | 21.91 |
| TCA | 514.28440 | 514.28381 | Negative | 30 | 5.48 |
| T-β-MCA | 514.28440 | 514.28381 | Negative | 30 | 2.58 |
| TDCA | 498.28948 | 498.28880 | Negative | 30 | 11.85 |
| TUDCA | 498.28948 | 498.28880 | Negative | 30 | 3.77 |
| TCDCA | 498.28948 | 498.28880 | Negative | 30 | 10.36 |
| dhCA（IS） | 401.23335 | 401.23288 | Negative | 20 | 1.71 |

**Supplementary Table 2** Regression equations, correlation coefficient, and linear ranges for individual bile acids in the Q-Exactive Focus Orbitrap MS method

| **Compound** | **Regression equations** | ***r*^2^** | **Linear range (ng/mL)** |
| --- | --- | --- | --- |
| CA | R=0.0185C+1.1453 | 0.9999 | 1.00~40000 |
| β-MCA | R=0.0361C+5.6804 | 0.9991 | 0.05~40000 |
| DCA | R=0.035C-1.539 | 0.9999 | 0.05~40000 |
| UDCA | R=0.0294C-0.5617 | 0.9999 | 0.01~40000 |
| CDCA | R=0.0273C+0.0476 | 0.9999 | 0.01~40000 |
| HDCA | R=0.031C+0.2011 | 0.9999 | 0.05~40000 |
| LCA | R=0.0589C+17.009 | 0.9960 | 0.01~40000 |
| TCA | R=0.0171C-6.3503 | 0.9948 | 0.05~40000 |
| T-β-MCA | R=0.0301C-7.2268 | 0.9972 | 0.01~40000 |
| TDCA | R=0.0261C-6.8886 | 0.9966 | 0.01~40000 |
| TUDCA | R=0.0254C-11.456 | 0.9945 | 0.10~40000 |
| TCDCA | R=0.021C-4.1966 | 0.9986 | 0.05~40000 |

**Supplementary Table 3** The primer sequences used for qRT-PCR.

| **Gene** | **Forward primer sequences (5’-3’)** | **Reverse primer sequences (5’-3’)** |
| --- | --- | --- |
| *Fxr* | AGGATAGAGAGGCAGTGGAGAAGC | AGCGTGGTGATGGTTGAATGTCC |
| *Ntcp* | ACCTACAAAGCTGCTGCAACTGAG | GGAAGGACCAGGTTGGAGAGGAG |
| *Bsep* | CGGCAACGCTCCAAGTCTCAG | GGGGCAGGTTCAACTTCTTCCAC |
| *Mrp2* | TGGATTCCCTTGGGCTTTCTTTGG | AACACGACGAACACCTGCTTGG |
| *β-actin* | TGTCACCAACTGGGACGATA | GGGGTGTTGAAGGTCTCAAA |
